# Poor sleep quality is negatively associated with low cognitive performance in general population independent of self-reported sleep disordered breathing

**DOI:** 10.1186/s12889-021-12417-w

**Published:** 2022-01-03

**Authors:** Zhongrong Wang, Mulalibieke Heizhati, Lin Wang, Mei Li, Zhikang Yang, Mengyue Lin, Reyila Abudereyimu, Jing Hong, Wenbo Yang, Ling Yao, Shasha Liu, Junli Hu, Nanfang Li

**Affiliations:** 1 Hypertension Center of People’s Hospital of Xinjiang Uygur Autonomous Region, Xinjiang Hypertension Institute National Health Committee Key Laboratory of Hypertension Clinical Research, No. 91, Tianchi Road Urumqi, Xinjiang, 830001 China; 2 Xinjiang Hypertension Institute, Xinjiang, China; 3 National Health Committee Key Laboratory of Hypertension Clinical Research, Xinjiang, China; 4 Key Laboratory of Xinjiang Uygur Autonomous Region “Hypertension Research Laboratory, Xinjiang, China; 5 Xinjiang Clinical Medical Research Center for Hypertension (Cardio-Cerebrovascular) Diseases, Xinjiang, China

**Keywords:** Sleep quality, Sleep disordered breathing, Low cognitive performance

## Abstract

**Background:**

Sleep disordered breathing (SDB) plays a significant role in both sleep quality and cognition and whether it has an impact on the relationship between above two factors remains to be clear. The study aimed to explore the association between sleep quality and cognitive performance in general population by considering influence of sleep disordered breathing (SDB).

**Methods:**

In this cross-sectional study, we enrolled subjects aged ≥ 18 years using a multi-stage random sampling method. Cognitive status was assessed using Mini Mental State Examination (MMSE) questionnaire, sleep quality using Pittsburgh Sleep Quality Index (PSQI) and SDB was assessed using No-SAS scale, respectively. Multi-variable logistic regression was applied to examine the association of sleep quality and cognitive performance. Subgroup analyses were performed in different age groups, and in those with and without SDB.

**Results:**

Finally, 30,872 participants aged 47.5 ± 13.8 years with 53.5% women were enrolled, of whom 32.4% had poor sleep quality and 18.6% had low cognitive performance. Compared with good sleepers, subjects with poor sleep quality exhibited significantly higher presence of low cognitive performance (23.7% vs 16.2%, P < 0.001). Poor sleepers revealed 1.26 (95%CI: 1.16,1.36), 1.26 (1.08,1.46) and 1.25 (1.14,1.37) fold odds for low cognitive performance in general population and in subjects with and without self-reported SDB respectively. Stratified by age and SDB, the association was observed in young and middle-aged group without SDB (OR = 1.44, 95%CI: 1.30,1.59) and in the elderly group with SDB (OR = 1.30, 95%CI: 1.07,1.58).

**Conclusions:**

Sleep quality is in a negative association with cognitive performance in general population independent of SDB, implying improvement of sleep disturbances is a potential objective of intervention strategies for cognitive protection at population level.

## Introduction

Cognitive impairment including dementia and Alzheimer’s disease (AD) has become one of the most serious public health problems worldwide [1, 2]. About 35.6 million people lived with dementia in 2010, with the number expected to double every 20 years [3]. Currently, there are no effective treatment options for the disease, leaving control of risk factors and prevention at pre-clinical stage as the effective way to delay disease onset [4].

Mild cognitive impairment (MCI), or low cognitive performance, is considered a transition stage between healthy cognitive aging and dementia, and can be identified years before dementia onset [5]. Nearly 2.4–42% population aged ≥ 60 suffer from MCI, based on studies from a number of countries using different screening and diagnostic cutoffs [6, 7]. Conversion rate from amnestic form of MCI to Alzheimer’s disease is estimated to be 10–15% per year [8].

Existing evidence suggests that poor sleep quality, per se a public health issue with a prevalence of 6%-94% in adults [9] be a risk factor for cognitive decline [10]. In Heinz Nixdorf Recall study, one of few existing relevant longitudinal studies, poor sleep quality, assessed using Pittsburgh Sleep Quality Index (PSQI), is associated with incident MCI in participants aged ≥ 50 years during mean follow-up 5.2 years [10]. In addition, in a meta analysis for cross-sectional and cohort studies of community dwellers, individuals with sleep problems including poor sleep quality show a pooled 1.68 times higher risk for combined outcome of cognitive impairment [11]. Furthermore, animal models indicate protective effects of better sleep quality on cognition by removal of neurotoxic waste products from central nervous system [12]. Nonetheless, results from studies are not always consistent, with some reporting null association between the two [13, 14]. The heterogeneity of results between these studies could be due to a number of methodological differences, including age [13–15] and sex [14] of participants, population culture or ethnicity [13, 15, 16], cognitive assessments or sleep measures [16], and statistical adjustments made for various potential confounders [13–15].

Importantly, effects of sleep disordered breathing (SDB) on the association of the two were not considered or not evaluated systematically in previous studies [13–15]. SDB is an umbrella term, with a prevalence of 24.0%-83.8% [17] and with OSA as the most frequent type [18]. OSA is a well-established risk factor for cognitive decline [19] and shows a strong association with AD [20], which could be explained by the fact that hypoxia plays a vital role and may contribute to increases in Aβ production, thereby facilitating AD pathogenesis [19]. Furthermore, Dlugaj et al. reported in small sample individuals aged 45–75 years that poor sleep quality is associated with low cognitive performance independent of apnea hypopnea index, a parameter of OSA severity [16]. Meanwhile, emerging evidence shows that sleep quality improvements can be achieved using cost-effective interventions and are beneficial in disease prevention [21]. Therefore, considering the high prevalence of SDB in general population, the well documented associations between SDB with sleep quality and cognitive performance, it might be of important significance to evaluate whether the relationship between sleep quality and low cognitive performance is independent of SDB, which may help public health interventions to formulate sleep quality improvement programs for cognitive protection at population level.

To our knowledge, there is few large-scale population-based epidemiological surveys to assess the association due to difficulty in assessing SDB in population-based studies. Current guidelines recommend polysomnography (PSG) as the gold standard for SDB assessment, which is an expensive time-consuming, labor-intensive, and technique dependent equipment, and inconvenient to be utilized in large scale population-based studies [22]. Nonetheless, NOSAS scale is a simple, efficient, and easy method for screening of self-reported SDB [23] and validated in some of Chinese population [24, 25] with a high sensitivity and specificity.

Therefore, the main objective of current article is to examine the association of overall sleep quality and low cognitive performance, by considering the influence of self-reported SDB in a large sample, representing the general adult population.

## Methods

### Site

Emin County, Northern Xinjiang China, is home to multi-ethnic population of over 160,000, living in urban, agricultural and stock-raising settings with higher prevalence of risk factors for cardiovascular disease with poor management [26, 27].

### Study population

In this cross-sectional study, we used multi-stage stratified sampling method to enroll study population aged ≥ 18 years. At the 1^st^ stage, Emin county was divided into urban, agricultural and stock raising settings as in our previous study [27]. At the 2^nd^ stage, two corresponding streets, villages or groups were selected using sample random sampling (SRS) method. At the 3^rd^ stage, subjects aged ≥ 18 years were selected using SRS method from the residence data. Inclusion criteria encompassed: 1. local inhabitants aged ≥ 18 years and residing at current address for ≥ 6 months; 2. agreement to participate and to sign an informed consent form. Exclusion criteria included confirmed dementia, confirmed AD, malignant tumors including those in central nervous system by computerized tomography and magnetic resonance imaging, encephalitis or meningitis, injury, trauma and or operations in central nervous system; those who are unable to co-operate due to mental reasons or others, and sever diseases on respiratory and or digestive system and night workers. To have higher response rate, survey was conducted together with annual health check up program between March to November 2019.

### Data collection

Each participant completed a questionnaire including PSQI, Mini Mental State Examination (MMSE), Global Physical Activity Questionnaire (GPAQ), Zung’s Self Rating Anxiety and Depression Scale (SAS, SDS) questionnaires. Data were also collected on participants’ demographic characteristics (age, gender, ethnicity), socioeconomic status (occupation, education status), lifestyles (cigarette and alcohol consumption) and medical histories (such as hypertension).

Trained staff measured body height, weight, neck circumference (NC), waist circumference (WC), and blood pressure (BP) of each participant using standardized protocol. Before BP measurement, each participant was asked to avoid drinking alcohol, smoking cigarettes and drinking coffee or tea. Each participant’s BP records were obtained using with OMRON HBP-1300 Professional Portable Blood Pressure Monitor (OMRON, Kyoto, Japan) three times on the right arm positioned at heart level with participant sitting at rest for five minutes, with 30 seconds between each measurement with an observer present. Average of three readings was used for analysis. Overnight fasting blood samples were obtained for measuring serum fasting blood glucose (FBG), lipid profiles and creatinine with clinical standard procedures at local hospitals.

### Assessment of cognitive status

Trained investigators evaluated cognitive status with MMSE [28], with a total score ranging from 0–30. Low cognitive performance is defined as MMSE score < 17, < 20 and < 24 for subjects with no formal education, 1–6 years of and with ≥ 7 years of education respectively [29].

### Assessment of sleep quality

Sleep quality was assessed with PSQI [30], the most used self-report instrument for assessing sleep quality of preceding 4 weeks. It was consisted with subjective sleep quality, sleep latency, sleep duration, sleep efficiency, sleep disturbances, use of sleep medication, and daytime dysfunction. A global score ranges from 0 to 21 and score ≥ 6 indicates poor sleep quality [31].

### Assessment of SDB

NoSAS score was used to assess self-reported SDB, score of which ranges from 0 to 17. self-reported SDB was defined with a NoSAS score ≥ 8 [23].

### Definitions of co-variables

Hypertension is defined as systolic BP ≥ 140 mmHg and/or diastolic BP ≥ 90 mmHg and/or receiving anti-hypertensive medication within previous 2 weeks [32]. Diabetes mellitus is defined as FBG ≥ 7.0 mmol/L, and/or self-reported previously diagnosed by physicians and/or intake of hypoglycemic agents within past 2 weeks [33]. Dyslipidemia is defined as total cholesterol ≥ 6.2 mmol/L and/or triglyceride ≥ 2.3 mmol/L and/or high density lipoprotein cholesterol < 1.0 mmol/L and/or low density lipoprotein cholesterol ≥ 4.2 mmol/L and/or having received treatment during past 2 weeks [34]. Coronary heart disease (CHD) and stroke were self-reported by the participants. Anemia was defined based on hemoglobin < 12 g/dL for men and < 11 g/dL for women [35]. Standard SAS and SDS score of 45 and 50 is set as cut-off for presence of anxiety and depression status respectively [36, 37]. Physical activity levels were grouped into high (≥ 3000 MET-minutes per week from any combination of walking, moderate-or vigorous-intensity activities or ≥ 1500 MET-minutes per week from vigorous intensity activity), moderate (not meeting the criteria for the high category, but achieving ≥ 600 MET-minutes per week) and low (not meeting any of the above criteria) categories [38]. Alcohol intake is defined as consuming alcoholic beverage at least once per week in the past month. Cigarette consumption is defined as participants who have smoked at least 20 packets of cigarettes in their lifetime and currently smoke cigarettes and non-smokers as participants who never smoked or smoked < 20 packets of cigarettes in their entire lifetime.

### Statistical analysis

Current study is a post-hoc analysis of the whole data collected in Emin in 2019. Students’ t-test was used to assess between-group differences in continuous variables if normally distributed; otherwise, nonparametric (Mann–Whitney U) test was applied. X^2^-test was used to assess between-group differences of categorical variables. P for trend was calculated by Kruskal–Wallis H test and X^2^ trend test for ordinal variables. Multiple logistic regression was used to estimate unadjusted and adjusted odds ratios (ORs) and 95% confidence intervals (95% CIs) for the association between poor sleep quality and low cognitive performance. Tolerance and variance inflation factor (VIF) were examined to identify multicollinearity and multicollinearity is a concern if VIF is > 10 and the tolerance is < 0.10 [39]. Before creating regression models, independent variables significantly relevant to low cognitive performance were selected using bi-variate analysis. Model a was adjusted for age and gender. Model b was adjusted for model a plus ethnicity, marital status, education attainment status, occupation, living setting, cigarette and alcohol consumption, BMI, physical activity level, estimated glomerular filtration rate (eGFR), anemia, anxiety and depression status, hypertension, dyslipidemia, diabetes mellitus, CHD and stroke. Model c was additionally adjusted for self-reported SDB. Age and or self-reported SDB was removed from the model when it was the stratification variable. Results were considered statistically significant if two-tailed p value was less than 0.05. All statistical analyses were performed with SPSS statistical software, version 24.0 (Chicago, IL, USA).

## Results

### Participants characteristics at baseline

Finally, a total of 30,872 participants with complete MMSE and PSQI data were comprised analytical sample, with an average age of 47.5 and 46.5% of men, of whom 32.4% suffered from poor sleep. Participants with poor sleep quality were older, having higher BMI, more likely to be women and take manual labor and less likely to smoke and drink, compared those with good sleep quality. Individuals with chronic diseases such as hypertension, dyslipidemia, diabetes mellitus, CHD, stroke, depression, anxiety and self-reported SDB were more likely to have poor sleep quality (Table 1).

**Table 1:** Characteristics of study population by sleep quality

	**Total (*n* = 30,872)**	**Good (*n* = 20,866)**	**Poor (*n* = 10,006)**	**t, X**^**2**^**, Z/P**
Gender (men,*n*,%)	14,347 (46.5)	10,728 (51.4)	3619 (36.2)	631.894/ < 0.001
Age (Mean ± SD, years)	47.5 ± 13.8	45.4 ± 13.4	51.8 ± 13.5	39.132/ < 0.001
Age group (*n*,%) ≤ 44	12,570 (40.7)	9797 (47.0)	2773 (27.7)	1323.068/ < 0.001
45–59	12,213 (39.6)	7891 (37.8)	4322 (43.2)	
≥ 60	6089 (19.7)	3178 (15.2)	2911 (29.1)	
Ethnicity (*n*,%), Han	15,318 (49.7)	10,051 (48.2)	5267 (52.7)	89.052/ < 0.001
Kazakh	10,236 (33.2)	7282 (34.9)	2954 (29.6)	
Others	5293 (17.2)	3520 (16.9)	1773 (17.7)	
Marital status (*n*,%) unmarried	2431 (7.9)	1927 (9.3)	504 (5.0)	441.202/ < 0.001
married	25,571 (83.0)	17,414 (83.7)	8157 (81.7)	
divorced/widowed	2790 (9.1)	1467 (7.1)	1323 (13.3)	
Education (*n*,%), ≤ primary	9858 (32.0)	5999 (28.8)	3859 (38.6)	305.939/ < 0.001
Junior high	11,716 (38.0)	8372 (40.2)	3344 (33.5)	
≥ Senior high	9247 (30.0)	6460 (31.0)	2787 (27.9)	
Region (*n*,%), urban	11,058 (36.2)	7023 (34.0)	4035 (40.8)	143.364/ < 0.001
stock-raising	3669 (12.0)	2652 (12.8)	1017 (10.3)	
agricultural	15,823 (51.8)	10,974 (53.1)	4849 (49.0)	
Occupation (*n*,%), mental labour	8898 (30.2)	6142 (30.9)	2756 (28.8)	14.186/ < 0.001
physical labor	20,519 (69.8)	13,704 (69.1)	6815 (71.2)	
Current smokers (*n*,%)	7748 (26.3)	5766 (28.8)	1982 (20.9)	207.420/ < 0.001
Current drinkers (*n*,%)	2807 (9.2)	2010 (9.7)	797 (8.0)	22.728/ < 0.001
Body mass index (Mean ± SD, kg/m^2^)	25.8 ± 4.3	25.7 ± 4.2	26.1 ± 4.4	8.059/ < 0.001
Body mass index ≥ 25 (*n*,%)	16,776 (54.9)	11,101 (53.7)	5675 (57.2)	33.796/ < 0.001
Systolic pressure (Median,quartile)	123 (111, 139)	122 (111, 137)	126 (114, 141)	14.317/ < 0.001
Diastolic pressure (Median,quartile)	79 (70, 88)	79 (70, 87)	80 (71, 88)	5.801/ < 0.001
Hypertension (*n*,%)	11,200 (36.3)	6816 (32.7)	4384 (43.8)	363.582/ < 0.001
Dyslipidemia (*n*,%)	9114 (29.6)	5414 (26.0)	3700 (37.1)	395.841/ < 0.001
Diabetes mellitus (*n*,%)	3147 (10.2)	1861 (8.9)	1286 (12.9)	114.166/ < 0.001
Coronary heart disease (*n*,%)	422 (1.4)	165 (0.8)	257 (2.6)	158.695/ < 0.001
Self-reported stroke (*n*,%)	1060 (3.5)	465 (2.2)	595 (6.0)	285.744/ < 0.001
Depression (*n*,%)	1202 (3.9)	332 (1.6)	870 (8.8)	916.267/ < 0.001
Anxiety (*n*,%)	2170 (7.1)	556 (2.7)	1614 (16.2)	1885.621/ < 0.001
NoSAS score (Median,quartile)	5 (2, 7)	5 (2, 7)	5 (3, 8)	15.820/ < 0.001
Sleep disordered breathing (*n*,%)	7265 (23.5)	4547 (21.8)	2718 (27.2)	108.469/ < 0.001
Physical activity (*n*,%), low	4213 (13.9)	2792 (13.7)	1421 (14.5)	8.377/0.015
moderate	8220 (27.2)	5500 (26.9)	2720 (27.7)	
high	17,823 (58.9)	12,160 (59.5)	5663 (57.8)	
Serum creatinine (Median,quartile)	69.2 (58.9, 81.0)	69.5 (59.4, 81.2)	68.2 (57.8, 80.8)	5.963/ < 0.001
eGFR (Median,quartile)	99.9 (83.8, 111.3)	102.1 (86.2, 113.1)	95.6 (78.8, 107.1)	23.266/ < 0.001
Total cholesterol (Median,quartile)	4.7 (4.0, 5.5)	4.7 (4.0, 5.5)	4.8 (4.1, 5.6)	11.683/ < 0.001
Triglyceride (Median,quartile)	1.2 (0.9, 1.8)	1.2 (0.8, 1.7)	1.3 (0.9, 1.8)	6.904/ < 0.001
Fasting blood glucose (Median,quartile)	5.3 (4.8, 5.9)	5.3 (4.8, 5.8)	5.4 (4.9, 6.0)	10.022/ < 0.001

*eGFR *estimated glomerular filtration rate

### MMSE scores and presence of low cognitive performance

**Fig. 1:**
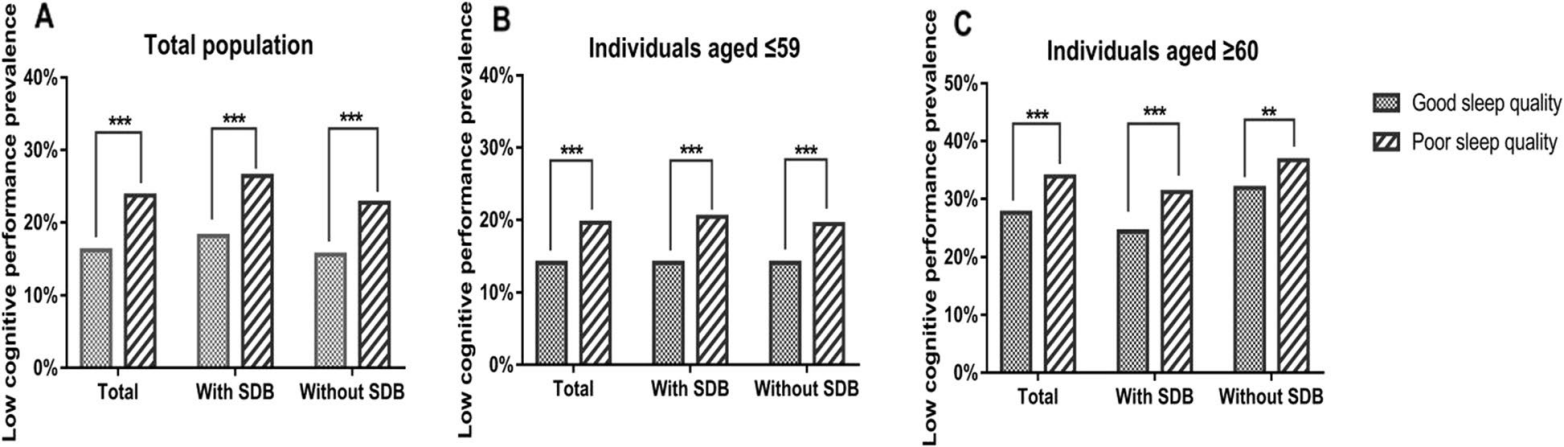
Prevalence of low cognitive performance by sleep quality in total population (**A**), individuals aged ≤ 59 (**B**) and ≥ 60 years (**C**), with and without SDB (*** and ** indicated statistical significance *P* ≤ 0.001 and ≤ 0.005, respectively.)

Median MMSE score was 26 and overall prevalence of low cognitive performance was 18.6% in total study participants. MMSE score was significantly lower in poor sleep quality group (25.0 vs 27.0, *P* < 0.001) and accordingly prevalence of low cognitive performance was significantly higher (23.7% vs 16.2%, *P* < 0.001), compared with the good sleep quality group. Similar results were observed in different age and self-reported SDB subgroups (Fig. 1).

### Association between sleep quality and low cognitive performance (Table 2)

**Table 2:** Associations between sleep quality and MCI by logistic regression models in total population and age subgroups by presence of *SDB*

	**Crude OR (95%CI), ** ***P***	**Adjusted** ^**a**^ ** OR(95%CI), ** ***P***	**Adjusted** ^**b**^ ** OR(95%CI), ** ***P***	**Adjusted** ^**c**^ ** OR(95%CI), ** ***P***
**Total population**				
Good sleep quality	Ref	Ref	Ref	Ref
Poor sleep quality	1.61 (1.52, 1.71), < 0.001	1.24 (1.17, 1.32), < 0.001	1.26 (1.16, 1.36), < 0.001	1.26 (1.16, 1.36), < 0.001
**Individuals with SDB**				
Good sleep quality	Ref	Ref	Ref	
Poor sleep quality	1.61 (1.44, 1.80), < 0.001	1.24 (1.10, 1.40), < 0.001	1.26 (1.08, 1.46), 0.003	
**Individuals without SDB**				
Good sleep quality	Ref	Ref	Ref	
Poor sleep quality	1.59 (1.48, 1.70), < 0.001	1.24 (1.16, 1.34), < 0.001	1.25 (1.14, 1.37), < 0.001	
**Population aged ≤ 59 years**				
Good sleep quality	Ref	Ref	Ref	Ref
Poor sleep quality	1.48 (1.38, 1.59), < 0.001	1.42 (1.32, 1.53), < 0.001	1.43 (1.30, 1.57), < 0.001	1.43 (1.30, 1.56), < 0.001
**Individuals with SDB**				
Good sleep quality	Ref	Ref	Ref	
Poor sleep quality	1.56 (1.31, 1.86), < 0.001	1.29 (1.08, 1.56), 0.006	1.26 (0.99, 1.59), 0.056	
**Individuals without SDB**				
Good sleep quality	Ref	Ref	Ref	
Poor sleep quality	1.46 (1.35, 1.58), < 0.001	1.42 (1.31, 1.54), < 0.001	1.44 (1.30, 1.59), < 0.001	
**Population aged ≥ 60 years**				
Good sleep quality	Ref	Ref	Ref	Ref
Poor sleep quality	1.34 (1.21, 1.50), < 0.001	1.24 (1.11, 1.39), < 0.001	1.16 (1.01, 1.33), 0.033	1.17 (1.02, 1.34), 0.029
**Individuals with SDB**				
Good sleep quality	Ref	Ref	Ref	
Poor sleep quality	1.41 (1.21, 1.64), < 0.001	1.30 (1.11, 1.52), 0.001	1.30 (1.07, 1.58), 0.008	
**Individuals without SDB**				
Good sleep quality	Ref	Ref	Ref	
Poor sleep quality	1.24 (1.06, 1.45), 0.008	1.19 (1.02, 1.40), 0.031	1.04 (0.85, 1.27), 0.705	

^a^adjusted for age, gender; ^b^model a plus ethnicity, marriage status, education status, occupation, region, cigarette consumption, alcohol intake, body mass index, physical activity level, hypertension, diabetes mellitus, dyslipidemia, stroke, coronary heart disease, estimated glomerular filtration rate, anemia, depression and anxiety status; ^c^model b plus sleep disordered breathing. Removing age when it was the stratification variable. *OR*, odds ratio *CI*, confidence interval *SDB*, sleep disordered breathing

Compared to the reference group of cognitively impaired individuals, odds for presence of low cognitive performance in poor sleep quality group was 1.26 (95%CI: 1.16, 1.36) in total population, 1.26 (95%CI: 1.08, 1.46) in individuals suffering from self-reported SDB and 1.25 (95%CI: 1.14, 1.37) in those free of self-reported SDB after adjusting relevant confounders. When stratified by age, poor sleep quality was also associated with presence of low cognitive performance in participants aged ≤ 59 (*OR* = 1.43, 95%CI: 1.30, 1.56) and those aged ≥ 60 (*OR* = 1.17, 95%CI: 1.02, 1.34). Nevertheless, the association only remained in individuals aged ≤ 59 without self-reported SDB (*OR* = 1.44, 95%CI: 1.30, 1.59) and those aged ≥ 60 with self-reported SDB (*OR* = 1.30, 95%CI: 1.07, 1.58) in cross stratification analysis.

## Discussion

Current investigation is one of few large-scale population-based studies to explore the relationship between sleep quality and cognitive performance by considering SDB as a confounder. Main results encompass: 1) As sleep quality deteriorates, MMSE total score declines and presence of low cognitive performance ascends progressively. 2) Sleep quality is negatively associated with presence of low cognitive performance in general population independent of self-reported SDB. 3) When stratified by age, the association is confined to individuals aged ≤ 59 years without self-reported SDB and those aged ≥ 60 years with self-reported SDB.

In support of current results, population-based prospective cohort studies in community dwellers report that poor sleepers harbour higher risk for developing low cognitive performance than their counterparts [10]. In addition, in a meta analysis of observational studies including cross-sectional and cohort studies of community dwellers aged > 60 years, individuals with sleep problems show higher risk for the combined outcome of cognitive impairment than those with good sleep [11]. However, effects of SDB on the association of sleep quality and cognitive performance in population-based studies have remained less investigated.

One of the differences of the current study with previous population-based ones is the inclusion of self-reported SDB as a variable. And we observed that sleep quality is negatively associated with low cognitive performance independent of SDB in total participants and in individuals aged ≤ 59 years, but dependent on SDB in those aged ≥ 60 years. In line with current results, Dlugaj et al. reported in a small sample individuals aged 45 to 75 years that poor sleep quality is associated with low cognitive performance independent of apnea hypopnea index, an indicator of OSA severity assessed objectively using PSG [16]. Furthermore, as previously reported, whether treatment of SDB with continuous positive airway pressure (CPAP) reverses cognition impairment remains unknown [40], whereas CPAP improves sleep quality [41]. Importantly, simple interventions for improving sleep quality such as sleep hygiene and chronobiotics can also delay the progress of cognitive impairment [21]. However, based on limited evidence, it is difficult to define that sleep quality and self-reported SDB are independently related with cognitive decline. Therefore, future studies are needed to explore the nature of relationships between different parameters of sleep and variables of cognition.

Inconsistent association of sleep quality, self-reported SDB and cognitive function in the elderly and young and middle-aged population has also been observed in a previous study [42]. Possible reasons for this phenomenon may include the following. Prevalence of both poor sleep quality and SDB increases with age and is highly prevalent in the elderly [43], as high as 62.4% [44] and 80% [45] respectively. This may blur the real association or effects of SDB on the association may weigh poor sleep quality. On the other hand, subjective assessment of changes in sleep quality using questionnaire like PSQI, may not be sensitive in the elderly. As an example, in a population-based study, generic complaints of sleep disturbances declined across the life span, with the highest in the aged 18–24 years, peaked again in the aged 45–59 years and the lowest in the elderly [46]. However, further studies are needed in this specific population group.

Several strengths merit this study as followings: First, this is one of the few large-scale population-based epidemiological studies to explore the association between sleep quality and cognitive performance involving SDB. Second, multiple confounders have been adjusted in the study such as self-reported SDB, depression, anxiety, eGFR, physical activity and so on, which are previously proven to be associated with low cognitive performance [47, 48], making the current results more reliable ones. Third, with a multi-stage random sampling method, participants in this survey are in a wide age range of men and women from general population and results can be applied to general population. However, a few limitations should also be considered, while explaining the data. Cross-sectional design makes it difficult to define causality and directionality of the association between sleep quality and low cognitive performance. Nonetheless, current results are in line with previous population-based cohort studies, in which baseline poor sleep quality is associated with cognitive decline during follow up [10]. Therefore, it may indicate current results are reliable. And we further extended previous results to those with and without self-reported SDB by considering the age. Second, we assessed subjective sleep quality using PSQI. Although it entails some subjectivity, PSQI is a more convenient method to identify poor sleep quality in practice than the objective but prohibitively expensive methods such as PSG [22]. Previous studies revealed that PSQI global score has moderate associations with some objective sleep quality indexes including PSG sleep maintenance, sleep efficiency, and microarousal index [31]. Considering the high internal consistency (α = 0.83), test- retest reliability (*r* = 0.85) [30], and moderate structural validity identifying subjects with poor sleep quality in both clinical and non-clinical populations [31], PSQI is a popular, reliable and valid instrument. Third, effectiveness of PSQI for evaluating sleep quality in population with low cognitive performance remains to be evaluated, which may have some bias on current results. Importantly, interventions for improving sleep quality have been shown to delay progress of cognitive impairment [21]. Fourth, one time assessment of cognitive function by MMSE is also a limitation. Combined with other tests of cognitive function (such as Montreal cognitive assessment) will help to make more augmented association with sleep quality. However, MMSE is a worldwide used measurement for cognitive screening [28] with the same cut-offs demonstrating high validity [49] and even suitable in younger population [50].

In conclusion, sleep quality is in a negative association with prevalent low cognitive performance in general population independent of self-reported SDB, implying improvement of sleep disturbances is a potential objective of intervention strategies for cognitive protection at population level.

## Data Availability

Data is available from corresponding author upon reasonable request.
